# Effects of Dietary Spirulina Supplementation on Cecal Microbiota, Serum Biochemistry, and Antioxidant Capacity in Lambs

**DOI:** 10.3390/microorganisms14020288

**Published:** 2026-01-27

**Authors:** Yuxuan Wang, Yushan Jia, Gentu Ge, Jian Bao, Xia Ding, Xiangdong Liu, Zhijun Wang

**Affiliations:** 1 Key Laboratory of Forage Cultivation, Processing and Highly Efficient Utilization of Ministry of Agriculture and Rural Affairs, College of Grassland Science, Inner Mongolia Agricultural University, Hohhot 010019, China; yx_wang7@163.com (Y.W.); jys_nm@sina.com (Y.J.); gegentu@163.com (G.G.); dingnmg@163.com (X.D.); 18847788933@163.com (X.L.); 2 Key Laboratory of Grassland Resources, Ministry of Education, College of Grassland Science, Inner Mongolia Agricultural University, Hohhot 010019, China; 3 College of Grassland Science, Inner Mongolia Agricultural University, Hohhot 010019, China; 4 Inner Mongolia Academy of Agricultural and Animal Husbandry Sciences, Hohhot 010031, China; 19940203@163.com; 5 College of Desert Control Science and Engineering, Inner Mongolia Agricultural University, Hohhot 010018, China

**Keywords:** Spirulina, lambs, cecal microbiota, serum parameters, antioxidant enzymes

## Abstract

Spirulina (*Limnospira platensis*) has gained attention as a functional feed additive in animal nutrition. However, its effects on the cecal microbiota and systemic metabolic responses in lambs remain unclear. Here, twelve 5-month-old male Hu lambs were randomly assigned to a control group (Control) or a Spirulina-supplemented group (SPI; 1.5% of dietary dry matter) and fed for 60 days (*n* = 6 per group). We measured serum biochemical indices, energy-metabolism variables, and immune and antioxidant parameters and characterized the cecal microbiota using 16S rRNA gene sequencing. Spirulina supplementation increased serum triglycerides (TG), glucose (GLU), alanine aminotransferase (ALT), and aspartate aminotransferase (AST) (*p* < 0.05). In the cecum, α-diversity indices were lower in SPI than in Control (*p* < 0.01), and Principal Coordinates Analysis (PCoA) indicated distinct community separation between groups (*p* < 0.01). Spearman correlation analyses further linked dominant genera to host metabolic and antioxidant traits. Collectively, Spirulina altered lamb metabolism and antioxidant status and reshaped the cecal microbial community, with microbial shifts associated with changes in serum indices.

## 1. Introduction

Spirulina (*Limnospira platensis*) is a protein-rich microalga increasingly used as a functional ingredient in animal feeds, largely due to its antioxidant and immunomodulatory bioactive compounds [1,2]. On a dry-matter basis, Spirulina typically contains ~60–75% crude protein, making it an attractive plant-based protein source. Its lignin-free cell wall (mainly peptidoglycan and polysaccharides) contributes to relatively low fiber contents (8–15% neutral detergent fiber, NDF; 5–10% acid detergent fiber, ADF). Spirulina also provides γ-linolenic acid, β-carotene, vitamin B12, chlorophyll, and phycocyanin, a blue pigment with strong antioxidant and anti-inflammatory activity that can scavenge free radicals and help protect cells from oxidative damage [3,4,5]. Accordingly, Spirulina supplementation has attracted increasing interest for improving metabolic traits and overall health in livestock.

The gastrointestinal microbiota is central to nutrient utilization and host metabolism in ruminants. While much of the research has focused on rumen function, the hindgut (including the cecum) is another important fermentation site where microbial metabolites interact with host energy and lipid metabolism [6]. Short-chain fatty acids produced by gut microbes are closely involved in regulating energy homeostasis and lipid metabolism [7]. Moreover, the cecal microbiota differs substantially from the rumen microbiota in composition and functional potential, underscoring the importance of hindgut microbiota in ruminant nutrition research [8,9,10]. In Hu sheep, cecal microbial profiles have been associated with fat deposition traits and serum biochemical parameters, suggesting that the cecum is a relevant target for nutritional regulation [11,12,13].

Previous studies in Hu sheep indicate that Spirulina supplementation can influence systemic metabolism and oxidative status. As a scalable source of antioxidant-rich compounds, microalgae such as Spirulina have been explored in ruminant feeding strategies. For example, Liang et al. found that 1% and 3% Spirulina supplementation alleviated high-energy diet-induced lipid metabolism disorders and oxidative stress while improving antioxidant capacity [14]. In addition, recent work in lambs has examined Spirulina effects on rumen microbiota and fermentation, showing that Spirulina can modulate rumen microbial ecology while altering host biochemical responses [15,16,17].

Mechanistically, Spirulina may affect lamb physiology through at least two complementary pathways. First, its fermentable components and putative prebiotic substrates can reshape the gastrointestinal microbial ecosystem. Dietary Spirulina has been reported to alter microbial community structure in both the rumen and cecum; for instance, 3% Spirulina increased the abundance of beneficial taxa such as butyrate-producing bacteria while suppressing potential pathogens in Hu sheep [15]. Second, Spirulina provides potent antioxidant compounds (e.g., phycocyanin, β-carotene, and polyphenols) that can directly scavenge reactive species and support endogenous antioxidant defenses, thereby mitigating oxidative stress–related cellular damage [18,19]. These observations highlight the cecum as an informative site for evaluating dietary interventions aimed at modulating metabolic homeostasis and oxidative status.

Therefore, this study evaluated whether dietary Spirulina supplementation in growing lambs modifies (i) serum biochemical and energy-metabolism-related parameters, (ii) antioxidant and humoral immune indices, and (iii) the diversity and composition of the cecal microbiota. We further tested whether variation in predominant cecal genera was associated with host serum indices. We hypothesized that Spirulina would reshape the cecal microbial community as well as alter host metabolic and antioxidant status, and that these microbial shifts would be associated with changes in serum parameters.

## 2. Materials and Methods

### 2.1. Animals and Experimental Design

The feeding trial was conducted from May to July 2023 at Baotou Lücao Animal Husbandry Development Co., Ltd. (Jiuyuan District, Baotou, China). During the study, daily ambient temperatures ranged from 17 to 24 °C, with peak temperatures of 34–38 °C, and relative humidity was ~60%. To reduce heat stress, pens were modified with open-sided designs and roof insulation, and cooling was provided using industrial fans combined with sprinkler systems.

The experiment lasted 75 days, including a 15-day adaptation period. Twelve healthy male Hu lambs (5 months old) with similar initial body weights (24.04 ± 1.25 kg; mean ± SD) were enrolled. Lambs were randomly assigned (random number generator) to a control group (Control; basal diet) or a Spirulina group (SPI; basal diet plus 1.5% Spirulina on a dry-matter basis), with each animal serving as an experimental unit (*n* = 6 per group). Animals were housed individually in pens (approximately 3 m × 4 m) equipped with individual feeders and automatic waterers, with ad libitum access to water throughout the trial.

### 2.2. Feed and Feeding Management

The basal diet was offered as a total mixed ration (TMR), with ingredients and analyzed nutrient composition shown in Table 1. Spirulina powder was obtained from Inner Mongolia Yike Biological Technology Co., Ltd. (Ordos, China). The proximate composition of the Spirulina powder was not determined for the specific batch used; therefore, the composition values cited in the Introduction are based on published reports and may vary across producers and batches. To ensure uniform intake, Spirulina powder was first premixed with the concentrate portion and then thoroughly incorporated into the TMR; mixing uniformity was checked daily before feeding. Lambs were fed twice daily (08:00 and 17:00), and feed was offered at 110% of the previous day’s intake. Refusals were minimal, indicating consistent consumption of the experimental diets. Cecal contents were collected for microbiota analysis upon slaughter on day 60 (see Section 2.5).

### 2.3. Chemical Analysis of Diets

Feed samples were dried at 65 °C for 48 h to determine initial dry matter (DM). Final DM was determined according to the AOAC method 930.15. Crude protein (CP) was measured using the Kjeldahl method (AOAC 984.13). Ether extract (EE) was determined by Soxhlet extraction (AOAC 920.39) [20]. Neutral detergent fiber (NDF) and acid detergent fiber (ADF) were analyzed using the filter-bag technique with an ANKOM2000 fiber analyzer (ANKOM Technology, Macedon, NY, USA) following Van Soest et al. (1991) [21].

### 2.4. Serum Parameters Analysis

At the end of the trial, blood and cecal samples were collected from all lambs (*n* = 6 per group). The cecum was selected because it is a major hindgut fermentation site with a dense microbial community that contributes to nutrient digestion, energy homeostasis, and systemic metabolic regulation. Prior to slaughter, approximately 20 mL of blood was collected by jugular venipuncture into vacuum tubes. Samples were kept on ice, centrifuged at 4 °C for 20 min to obtain serum, and stored at −80 °C until analysis [22,23]. Serum TG, urea (UREA), GLU, NEFA, total protein (TP), total cholesterol (TC), and insulin (INS) were measured using routine enzymatic methods as described previously [24]. ALT and AST activities and HDL-C and LDL-C concentrations were determined by colorimetric assays [25]. Serum albumin (ALB), MDA, GSH-PX, SOD, CAT, IgA, IgG, and IgM were quantified using commercial kits (Nanjing Jiancheng Bioengineering Institute, Nanjing, China) following the manufacturer’s instructions [26,27,28].

### 2.5. Cecal Sample Collection, Genomic DNA Extraction, Amplification and Sequencing of 16S rRNA Genes

Cecal contents were collected aseptically immediately after slaughter and transferred to sterile polypropylene tubes. Samples were flash-frozen in liquid nitrogen and stored at −80 °C until analysis. Total genomic DNA was extracted using an Omega Bio-tek DNA kit (D4015, Norwalk, CT, USA) according to the manufacturer’s protocol. DNA concentration and purity were assessed using a NanoDrop 2000 spectrophotometer (Thermo Fisher Scientific, Wilmington, DE, USA), and integrity was confirmed by 2% agarose gel electrophoresis [29].

The V3–V4 regions of the 16S rRNA gene were amplified using primers 338F/806R. Sequencing was performed on an Illumina platform by Majorbio Biomedical Technology Co., Ltd. (Shanghai, China). Paired-end reads were merged using FLASH (v1.2.8), quality-filtered with fqtrim (v0.94), and screened for chimeras with VSEARCH (v2.3.4). Denoising and amplicon sequence variant (ASV) inference were conducted with DADA2 in QIIME2 (v2019.7). Taxonomic assignment was performed using the q2-feature-classifier against the SILVA v138 database. Community visualization and downstream analyses were conducted using the Majorbio I-Sanger platform (https://cloud.majorbio.com; accessed on 15 September 2023). Raw sequencing data have been deposited in the NCBI SRA under accession PRJNA1397909.

### 2.6. Statistical Analysis

Statistical analyses were conducted using six biological replicates per group (*n* = 6). Data normality was assessed using the Shapiro–Wilk test. Serum biochemical, immunological, and antioxidant parameters were compared using independent-samples *t*-tests, with Welch’s correction applied when variances were unequal. Data are presented as mean ± SD, and *p* < 0.05 was considered significant. For cecal α-diversity indices (observed ASVs, Chao1, Shannon, and Good’s coverage), Wilcoxon rank-sum tests were used. For β-diversity, Bray–Curtis distances were calculated and visualized by principal coordinates analysis (PCoA); group effects were tested using PERMANOVA. Differential taxa at the phylum and genus levels were identified using Wilcoxon rank-sum tests with Benjamini–Hochberg false discovery rate (FDR) correction. Associations between serum parameters and prevalent genera were evaluated by Spearman correlation and visualized as heatmaps.

## 3. Results

### 3.1. Serum Biochemical Indices

Table 2 summarizes the effects of dietary Spirulina supplementation on serum biochemical parameters. Compared with Control, lambs in the SPI group showed higher serum TG, ALT, AST, and GLU concentrations (*p* < 0.0001).

No significant differences were observed between groups for UREA, TP, ALB, TC, HDL-C, or LDL-C (*p* > 0.05).

### 3.2. Energy Metabolism, Immune Response, and Antioxidant Status

As shown in Table 3, Spirulina supplementation significantly affected several energy-metabolism parameters. Compared with Control, SPI lambs had lower NEFA concentrations and higher INS levels (*p* < 0.01). Spirulina also increased the activities of SOD and CAT (*p* < 0.01), whereas MDA concentration and GSH-PX activity did not differ between groups (*p* > 0.05). Serum IgA, IgG, and IgM concentrations were not significantly affected (*p* > 0.05).

### 3.3. Spirulina Supplementation Reshapes the Cecal Microbial Community Structure in Lambs

Sequencing statistics and α-diversity indices are presented in Table 4. The total and valid sequence counts did not differ between groups (*p* > 0.05), and Good’s coverage exceeded 0.99 in both groups, indicating sufficient sequencing depth.

Microbial richness and diversity were lower in SPI than in Control. Observed ASVs and the Chao1 estimator were reduced in SPI (*p* < 0.01), and the Shannon index was also lower (*p* < 0.001).

**Figure 1:**
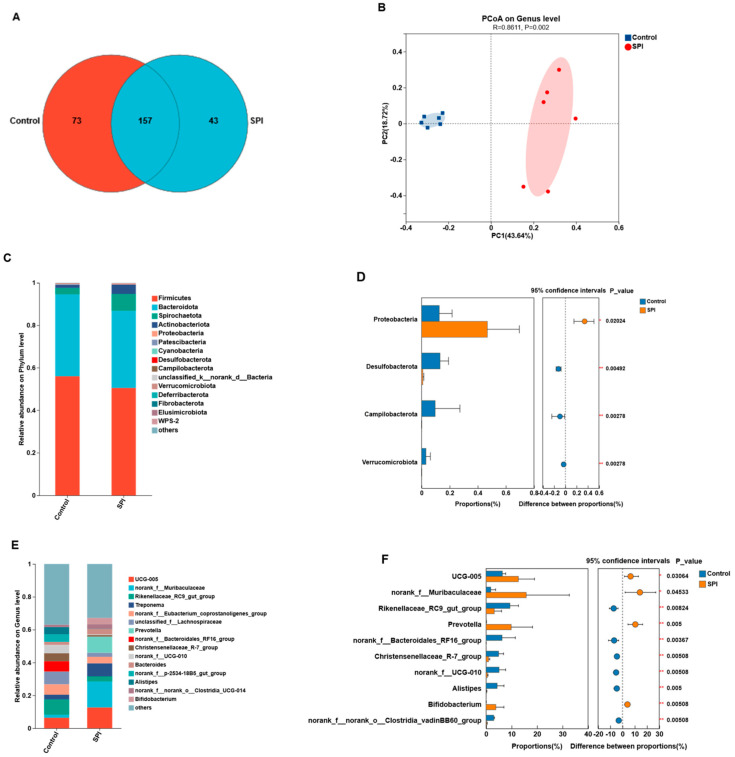
Bacterial community diversity and composition of lamb cecal samples. (**A**) Venn diagram showing the unique and shared ASVs between experimental groups. (**B**) Principal coordinate analysis (PCoA) of cecal bacterial communities based on Bray–Curtis distances. (**C**) Relative abundance (%) of bacterial phyla, showing the top 15 phyla ranked by relative abundance. (**D**) Differentially abundant phyla between experimental groups identified using the Wilcoxon rank-sum test. (**E**) Relative abundance (%) of bacterial genera, showing the top 15 genera ranked by relative abundance. (**F**) Differentially abundant genera between experimental groups identified using the Wilcoxon rank-sum test. Significance is indicated by * and ** for *p* < 0.05 and *p* < 0.01, respectively.

Venn analysis identified 157 shared ASVs, with 73 and 43 ASVs unique to the Control and SPI groups, respectively (Figure 1A). PCoA based on Bray–Curtis distances showed clear separation of samples by diet (PERMANOVA, *p* < 0.01; Figure 1B).

At the phylum level, Firmicutes and *Bacteroidota* were dominant in both groups (Figure 1C). Differential analysis indicated that *Proteobacteria* was enriched in SPI (*p* = 0.02024), whereas *Desulfobacterota*, *Campilobacterota*, and *Verrucomicrobiota* were more abundant in Control (*p* < 0.01; Figure 1D).

At the genus level, SPI increased the relative abundances of UCG-005, norank_f__Muribaculaceae, *Prevotella*, and *Bifidobacterium* (*p* < 0.05). Conversely, Rikenellaceae_RC9_gut_group, norank_f__Bacteroidales_RF16_group, Christensenellaceae_R-7_group, norank_f__UCG-010, *Alistipes*, and norank_f__norank_o__Clostridia_vadinBB60_group were more abundant in Control (*p* < 0.01; Figure 1F).

### 3.4. Correlation Heatmap of Predominant Cecal Bacteria and Serum Parameters

**Figure 2:**
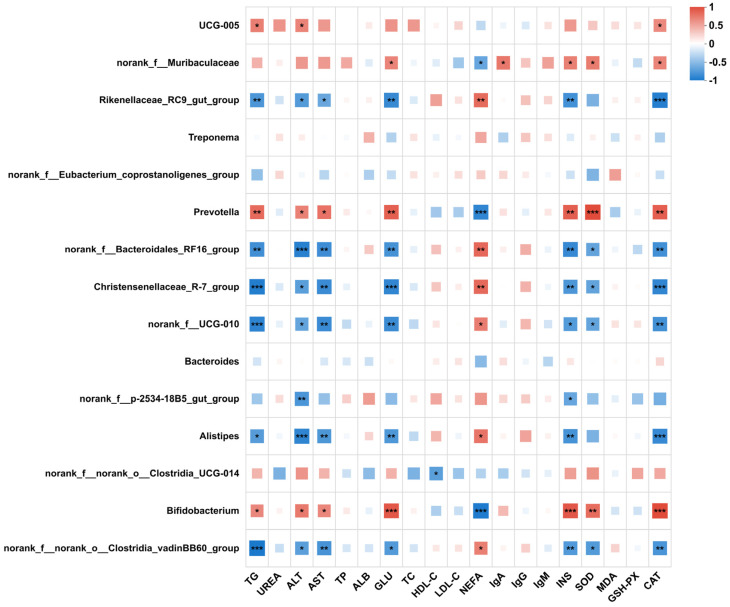
Spearman correlation heatmap between predominant cecal genera and serum parameters in lambs. Red indicates positive correlations and blue indicates negative correlations; color intensity reflects correlation strength (*ρ*). Significance is indicated by *, **, and *** for *p* < 0.05, *p* < 0.01, and *p* < 0.001, respectively.

Spearman correlation analysis was performed to examine relationships between predominant cecal genera and serum biochemical, metabolic, and antioxidant indices (Figure 2). *Prevotella* and *Bifidobacterium* were positively correlated with TG, ALT, AST, GLU, and INS, and also showed positive associations with SOD and CAT activities. Both genera were negatively correlated with LDL-C (*p* < 0.05).

In contrast, Rikenellaceae_RC9_gut_group, norank_f__Bacteroidales_RF16_group, Christensenellaceae_R-7_group, norank_f__UCG-010, and *Alistipes* were generally negatively correlated with TG, ALT, AST, GLU, INS, and CAT, while showing positive correlations with NEFA (*p* < 0.05). Overall, significant associations were concentrated in indices related to energy metabolism and antioxidant activity.

## 4. Discussion

Spirulina is rich in nutrients and bioactive compounds, and interest in its use as a functional feed additive has increased in recent years [30,31]. In the present study, Spirulina supplementation significantly altered serum metabolic profiles, antioxidant enzyme activities, and the composition of the cecal microbiota in growing lambs, highlighting coordinated host–microbiota responses to this dietary intervention.

Spirulina increased serum TG, GLU, ALT, and AST and was accompanied by higher INS and lower NEFA concentrations. This pattern indicates pronounced alterations in glucose and lipid metabolism, characterized by significantly higher circulating glucose and insulin levels [14,32]. Spirulina contains highly digestible protein, polysaccharides, and diverse bioactives that have been reported to influence insulin secretion and carbohydrate metabolism [33,34]. The lower NEFA levels further support enhanced antilipolytic insulin action [35,36].

In the present study, ALT (38.05 U/L) and AST (206.67 U/L) in the Spirulina group were slightly higher than the ranges reported by Očenáš et al. (2025) [37] and Zhao et al. (2025) [38]. While the serum activities of ALT and AST remained within or near the upper physiological limits reported for healthy lambs, the marked increase (*p* < 0.0001) compared to the Control group suggests a heightened metabolic load on the liver. Notably, serum total protein (TP) and albumin (ALB) remained stable and within expected biological ranges, and MDA did not change, suggesting no marked lipid peroxidation or membrane damage. Moreover, the concurrent increase in insulin and decrease in NEFA indicate a regulated shift in substrate utilization rather than uncontrolled hyperglycemia. Previous studies also suggest that Spirulina may modulate aminotransferase activity and improve hepatic lipid deposition and redox balance [39,40]. For example, in a high-fat diet–induced NAFLD rat model, spirulina peptide–loaded nanoliposomes reduced hepatic lipid accumulation and improved redox homeostasis through AMPK signaling [41]. Similarly, spirulina supplementation in sheep fed high-energy diets improved fatty acid metabolism and regulated autophagy-related gene expression, thereby promoting hepatic lipid homeostasis [14,32]. Collectively, these findings support a potential hepatoprotective role of Spirulina rather than overt hepatotoxicity. Therefore, the elevated ALT and AST observed here may reflect adaptive metabolic responses to dietary intervention; however, contributions from subclinical, reversible hepatocellular alterations or extrahepatic sources cannot be excluded. The absence of liver histology and additional hepatobiliary markers limits definitive interpretation.

Spirulina supplementation increased SOD and CAT activities, indicating an enhanced enzymatic antioxidant defense. In contrast, MDA concentration and GSH-PX activity were unchanged, suggesting that Spirulina primarily up-regulated selected antioxidant enzymes rather than broadly reducing lipid peroxidation under the present conditions [18,42]. Consistent patterns have been reported in other animal models, where Spirulina increased SOD and CAT but showed variable effects on glutathione peroxidase [43,44]. Serum immunoglobulin concentrations were not affected, implying that systemic humoral immunity remained stable during the trial. Previous evidence suggests that Spirulina’s immunomodulatory effects may be more apparent under immune challenge or at mucosal sites rather than as changes in circulating immunoglobulins in healthy animals [45,46].

Spirulina supplementation also reshaped the cecal microbial community, as evidenced by reduced richness and diversity and a clear separation between groups in β-diversity analyses. The Spirulina-supplemented group exhibited a significant reduction in microbial richness and α-diversity, with the Shannon index decreasing from 5.53 to 4.11. Such a contraction in community diversity often reflects the selective dominance of specific taxa, such as *Prevotella* and *Bifidobacterium*. Although decreased α-diversity is often interpreted as dysbiosis, diet-induced reductions can also reflect selective enrichment of taxa best adapted to newly available substrates, resulting in a more specialized community [47,48]. Similar reductions in α-diversity accompanied by distinct β-diversity clustering have been reported following Spirulina supplementation in other studies [49,50,51]. Given that Spirulina provides high-quality protein, γ-linolenic acid, phycocyanin, and diverse polysaccharides [48,52], it may supply specific substrates that confer a growth advantage to microbes capable of utilizing them, thereby driving the community toward a functionally specialized configuration [53,54]. While these shifts correlated with improved antioxidant enzyme activities, the loss of microbial diversity could potentially impact the long-term resilience and stability of the hindgut ecosystem. Therefore, this reshaped community should be viewed as a highly specialized configuration whose ecological implications require further investigation.

Correlation analysis further highlighted links between the remodeled cecal microbiota and host physiological status. In particular, the increased abundance of genera such as *Prevotella* and *Bifidobacterium* was positively associated with serum GLU and INS and with antioxidant enzyme activities (SOD and CAT) and negatively associated with NEFA. Together, these patterns suggest that Spirulina-associated microbial shifts are coupled with a metabolic transition from lipid mobilization toward greater glucose utilization and enhanced enzymatic antioxidant defense. These associations support the presence of a responsive microbiota–metabolism axis through which the cecal community may influence systemic energy partitioning and redox balance. Although correlation does not imply causation, our results are consistent with accumulating evidence that diet-driven microbial specialization is linked to host metabolic regulation [55,56].

## 5. Conclusions

Dietary Spirulina supplementation (1.5% of DM) showed a combination of positive and concerning effects in growing lambs. On the positive side, the treatment successfully boosted the host’s antioxidant defenses (increased serum SOD and CAT) and improved energy use efficiency (higher INS and lower NEFA levels). It also helped enrich beneficial gut bacteria like Prevotella and Bifidobacterium.

On the other hand, certain results suggest potential downsides. The significant rise in serum glucose, ALT, and AST, combined with the drop in microbial diversity, may indicate increased metabolic stress on the liver and a less stable gut environment. At this stage, it is not yet clear whether these changes represent a high-efficiency adaptation or a negative strain on the animal’s health. Therefore, while Spirulina shows potential, we cannot reach a final conclusion regarding its overall safety. Further research includes liver tissue examinations and longer feeding trials.

## Figures and Tables

**Table 1:** Ingredient composition and analyzed nutrient levels of the basal diet.

Item	Control	SPI
Ingredients (%)		
Forage		
Alfalfa	16.1	16.1
Concentrate		
Maize	42.63	42.63
Soybean meal	19.6	19.6
Flax	7.84	7.84
Wheat bran	5.88	5.88
Additives		
Premix	5	5
Salt	0.98	0.98
Soda	1.17	1.17
Spirulina	0	1.5
Chinese herbal mixture		
Hawthorn	0.1	0.1
Malt	0.1	0.1
Dried tangerine peel	0.1	0.1
Medicated Leaven	0.1	0.1
Astragalus membranaceus	0.1	0.1
Atractylodes macrocephala	0.1	0.1
Licorice	0.1	0.1
Epimedium	0.1	0.1
Chemical Compositions		
Dry matter (%)	65.78	66.91
Crude protein (%DM)	18.17	19.29
Acid detergent fiber (%DM)	15.98	15.70
Neutral detergent fiber (%DM)	23.51	24.86
Ether extract (%DM)	3.50	3.61
Metabolizable Energy (MJ/kg)	10.55	10.62

The 5% premix provided the following per kg of diet: Vitamin A 5000–10,000 IU, Vitamin D 2000–5000 IU, Vitamin E ≥ 25 mg, Nicotinic acid ≥ 30 mg, Cu 14–25 mg, Fe 100–200 mg, Zn 80–185 mg, Mn 40–80 mg, Ca 0.5–1.25%, and P ≥ 0.025%.

**Table 2:** Effects of Spirulina supplementation on serum biochemical parameters in lambs.

Item	Control	SPI	*p*-Value
TG (mmol/L)	0.15 ± 0.02 b	0.43 ± 0.01 a	<0.0001
Urea (mmol/L)	2.93 ± 0.10	3.04 ± 0.20	0.6412
ALT (U/L)	10.57 ± 0.91 b	38.05 ± 1.05 a	<0.0001
AST (U/L)	81.97 ± 3.97 b	206.67 ± 6.31 a	<0.0001
TP (g/L)	58.82 ± 0.93	59.69 ± 1.04	0.5471
ALB (g/L)	20.55 ± 0.47	20.05 ± 0.95	0.6493
GLU (mmol/L)	5.33 ± 0.31 b	14.12 ± 0.65 a	<0.0001
TC (mmol/L)	1.00 ± 0.09	1.10 ± 0.09	0.4385
HDL-C (mmol/L)	0.52 ± 0.02	0.51 ± 0.01	0.4901
LDL-C (mmol/L)	0.32 ± 0.05	0.29 ± 0.02	0.6735

TG, triacylglycerol; ALT, alanine aminotransferase; AST, aspartate aminotransferase; TP, total protein; ALB, albumin; GLU, glucose; TC, total cholesterol; HDL-C, high-density lipoprotein cholesterol; LDL-C, low-density lipoprotein cholesterol. Data are presented as mean ± SD. Different lowercase letters within a row indicate significant differences between experimental groups (*p* < 0.05). *n* = 6 per group.

**Table 3:** Effects of Spirulina supplementation on energy metabolism, immune indices, and antioxidant status in lambs.

Item	Control	SPI	*p*-Value
NEFA (mmol/L)	0.43 ± 0.01 a	0.38 ± 0.01 b	0.0005
IgA (g/L)	0.33 ± 0.01	0.33 ± 0.01	0.5859
IgG (g/L)	9.31 ± 0.40	8.92 ± 0.32	0.469
IgM (g/L)	0.58 ± 0.01	0.60 ± 0.01	0.1745
INS (μIU/mL)	10.82 ± 0.31 b	13.43 ± 0.31 a	0.0001
SOD (U/mL)	80.91 ± 0.57 b	84.39 ± 0.51 a	0.0011
MDA (nmol/mL)	3.01 ± 0.05	2.97 ± 0.06	0.6099
GSH-PX (U/mL)	189.30 ± 1.44	190.39 ± 1.66	0.6311
CAT (U/mL)	42.24 ± 0.40 b	48.27 ± 0.33 a	<0.0001

NEFA, non-esterified fatty acids; IgA, immunoglobulin A; IgG, immunoglobulin G; IgM, immunoglobulin M; INS, insulin; SOD, superoxide dismutase; MDA, malondialdehyde; GSH-PX, glutathione peroxidase; CAT, catalase. Data are presented as mean ± SD. Different lowercase letters within a row indicate significant differences between experimental groups (*p* < 0.05). *n* = 6 per group.

**Table 4:** Effects of Spirulina supplementation on diversity indices of the cecal microbial community in lambs.

Item	Control	SPI	*p*-Value
No. of sequences	73,171.83 ± 2799.79	80,158.83 ± 3581.10	0.155
No. of valid sequences	49,843.33 ± 1181.88	49,562.50 ± 1972.61	0.905
Observed ASV number	924.50 ± 107.98	442.17 ± 30.66	0.002
Chao1	951.52 ± 116.90	456.33 ± 33.96	0.002
Shannon	5.53 ± 0.17	4.11 ± 0.06	<0.001
Good’s coverage	0.9977 ± 0.0007	0.9987 ± 0.0003	0.192

Total No. of sequences across all samples = 919,984; total No. of valid sequences across all samples = 596,435. *n* = 6 per group.

## Data Availability

The original contributions presented in the study are included in the article, further inquiries can be directed to the corresponding author. The raw sequencing data have been deposited in the NCBI SRA under accession PRJNA1397909.

## Abbreviations

The following abbreviations are used in this manuscript:

Control	control group
SPI	Spirulina group
DM	Dry matter
TMR	Total mixed ration
CP	Crude protein
NDF	Neutral detergent fiber
ADF	Acid detergent fiber
TG	Triacylglycerol
UREA	Urea
GLU	Glucose
NEFA	Non-esterified fatty acids
TP	Total protein
ALB	Albumin
TC	Total cholesterol
HDL-C	High-density lipoprotein cholesterol
LDL-C	Low-density lipoprotein cholesterol
INS	Insulin
ALT	Alanine aminotransferase
AST	Aspartate aminotransferase
SOD	Superoxide dismutase
CAT	Catalase
MDA	Malondialdehyde
GSH-PX	Glutathione peroxidase
IgA	Immunoglobulin A
IgG	Immunoglobulin G
IgM	Immunoglobulin M
PCoA	Principal coordinate analysis
SRA	Sequence Read Archive
